# Lack of H^+^-pyrophosphatase Prompts Developmental Damage in *Arabidopsis* Leaves on Ammonia-Free Culture Medium

**DOI:** 10.3389/fpls.2016.00819

**Published:** 2016-06-10

**Authors:** Mayu Fukuda, Shoji Segami, Takaaki Tomoyama, Mariko Asaoka, Yoichi Nakanishi, Shizuka Gunji, Ali Ferjani, Masayoshi Maeshima

**Affiliations:** ^1^Laboratory of Cell Dynamics, Graduate School of Bioagricultural Sciences, Nagoya UniversityNagoya, Japan; ^2^Department of Biology, Tokyo Gakugei University, KoganeiTokyo, Japan

**Keywords:** *Arabidopsis thaliana*, H^+^-pyrophosphatase, pyrophosphate, proton pump, vacuole, plant growth

## Abstract

The plant vacuolar H^+^-pyrophosphatase (H^+^-PPase) functions as a proton pump coupled with the hydrolysis of pyrophosphate (PPi). Loss-of-function mutants (*fugu5*s and *vhp1*) of the H^+^-PPase of *Arabidopsis thaliana* show clear morphological phenotypes in the cotyledons, caused by inhibition of gluconeogenesis from seed storage lipids due to excessive accumulation of PPi. In this study, we investigated the phenotypes of the *fugu5* and *vhp1* mutants during vegetative growth under a specific nitrogen nutritional regime. When nitrate in the culture medium was the sole nitrogen source, growth of the mutant rosette leaves was severely compromised. Interestingly, trypan blue staining revealed notable cell death at the leaf blade–petiole junctions of young leaves, a region known to have meristematic features. Physical contact of the leaf tip with the culture medium also triggered leaf atrophy, suggesting that absorption of some elements through the hydathodes was probably involved in this phenotype. Prevention of such leaf–medium contact resulted in a marked decrease in phosphate content in the shoots, and suppressed leaf atrophy. Furthermore, *fugu5* necrotic symptoms were rescued completely by heterologous expression of yeast cytosolic soluble pyrophosphatase IPP1 or uncoupling-type H^+^-PPases that retained only PPi-hydrolysis activity, indicating that the damage of actively proliferating cells was caused by the loss of the PPi-hydrolyzing function of H^+^-PPase. Importantly, cell death and growth defects of the *fugu5* leaves were suppressed completely by the simple addition of ammonium (>1 mM) to the culture medium. The PPi content in the shoots of *fugu5* grown on ammonium-free medium was 70% higher than that of the wild type, and PPi levels were restored to normal upon growth on ammonium-supplemented medium. Together, these findings suggest that the PPi-hydrolyzing activity of H^+^-PPase is essential to maintain the PPi contents at optimal levels when grown on ammonium-free culture medium, and any direct contact of the leaves with the culture medium may raise PPi levels in the leaves through increased phosphate uptake.

## Introduction

Pyrophosphate, a high-energy phosphate compound, is generated by various metabolic processes, including macromolecule biosynthesis. For example, reactions catalyzed by DNA polymerase, RNA polymerase, aminoacyl-tRNA synthetase (protein synthesis), UDP-glucose pyrophosphorylase (UGPase; synthesis of cellulose and sucrose), and fatty acyl-CoA synthetase utilize nucleoside triphosphates as the substrate and release PPi as the byproduct. These metabolic reactions are particularly active, for example, in young plant tissues and organs with proliferating cells, and in germinating seeds (Maeshima, 2000; Heinonen, 2001; Ferjani et al., 2014a,b). In total, nearly 200 enzymatic reactions are known to release PPi (Heinonen, 2001). The PPi produced is then hydrolyzed by soluble-type pyrophosphatases (PPases) and vacuolar H^+^-PPases, to sustain the activities of PPi-generating reactions in plant cells. Whereas the soluble PPases hydrolyze PPi and dissipate the energy in the form of heat, H^+^-PPases efficiently utilize the energy released from PPi hydrolysis for the active translocation of protons into the vacuole (Maeshima, 2000; Martinoia et al., 2007). PPi is also essential for PFP (PPi-fructose 6-phosphate 1-phosphotransferase, EC 2.7.1.90; PFP), UGPase (glucosyl-1-phosphate uridyl transferase, EC 2.7.7.9), and pyruvate orthophosphate dikinase (pyruvate, phosphate dikinase, EC 2.7.9.1; PPDK) as a phosphoryl donor (Heinonen, 2001; Ferjani et al., 2014a,b). Therefore, the cytosolic concentration of PPi should be maintained at an adequate level to ensure optimal cellular functions. This maintenance might be achieved by fine-tuning the abundance or activities of H^+^-PPase and soluble PPases.

Previous studies have characterized the biochemical and physiological properties of H^+^-PPase (Maeshima, 2000; Gaxiola et al., 2007; Martinoia et al., 2007), but information on soluble PPases is scarce (Schulze et al., 2004; George et al., 2010; Öztürk et al., 2014). Vacuolar (type I) H^+^-PPase is highly abundant and accounts for more than 10% of the total amount of tonoplast proteins in young tissues (Maeshima, 2001). Type II H^+^-PPase is localized in the Golgi apparatus and the trans-Golgi network, and amounts to less than 0.3% of that of the type I enzyme in *Arabidopsis thaliana* (hereafter, *Arabidopsis*) (Segami et al., 2010). In this study, we investigated the loss-of-function mutants of the vacuolar H^+^-PPase to understand its physiological role in PPi homeostasis and plant growth.

The knockout mutant *avp1-1* of H^+^-PPase was reported to show severe growth defects in *Arabidopsis* (Li et al., 2005). Other groups independently reported that the H^+^-PPase mutant showed milder growth and developmental defects that were specific to the cotyledon and hypocotyl, but only on sucrose-free culture medium, with no noticeable developmental defect in other organs or at any other developmental stage (Ferjani et al., 2011; Segami et al., 2014). Indeed, functional analyses of the loss-of-function *fugu5* mutant series showed clearly that PPi hydrolysis is essential for active gluconeogenesis and sucrose synthesis *de novo* to sustain postgerminative growth of *Arabidopsis* seedlings (Ferjani et al., 2011).

In this study, we investigated the morphological properties of the *fugu5* mutants to understand the contribution of vacuolar H^+^-PPase to the early stages of vegetative growth, and to PPi homeostasis, in *Arabidopsis*. We focused on the composition of the culture medium and determined that different nitrogen sources affected the growth of *fugu5* mutants. Overall growth delay and severe atrophy of the rosette leaves were observed in the mutants when grown on ammonium-free culture medium. Importantly, such phenotypes were restored fully when ammonium was supplied. Careful observations suggested that physical contact of the mutant leaves with the ammonium-free culture medium was likely the trigger for leaf atrophy. Interestingly, the severely atrophic phenotypes in leaves were not observed in transgenic plants expressing a yeast-soluble PPase (IPP1) or uncoupling-type H^+^-PPases, which retained PPi-hydrolysis activity, but not proton pump activity, in the *fugu5* background. Finally, the significance of the PPi hydrolysis function of H^+^-PPase and its contribution to plant growth are discussed based on our current findings.

## Materials and Methods

### Plant Materials and Growth Conditions

Seeds of *Arabidopsis thaliana* (strain, Columbia-0) provided by the RIKEN Bioresource Center (Tsukuba, Japan) were surface sterilized and sown on sterile gel plates containing half-strength Murashige-Skoog (MS) salt mixture (Wako Pure Chemical, Osaka, Japan), 2.5 mM MES-KOH (pH 5.7), 2% (w/v) sucrose, and 0.5% gellan gum (MS plates). Seed specimens were placed in darkness at 4°C for 2 days, and then moved to a growth chamber at 22°C under long-day conditions (light/dark regime of 16 h/8 h, cool-white lamps, 90 μmol/m^2^ s). In addition to the wild type (WT), three loss-of-function mutant alleles of H^+^-PPase, also in the Col-0 background, were characterized. The *fugu5-1* (Ala-709 to Thr) mutant consists of a single amino-acid substitution and the *fugu5-3* line has an amino-acid substitution (Ala-533 to Thr) and the deletion of five residues (554–558) (Ferjani et al., 2007, 2011). A T-DNA insertion mutant of H^+^-PPase, *vhp1-1* (Kazusa DNA Research Institute; reference no. KG8420), was also used (Segami et al., 2014). The *vhp1-1* line was selected and characterized by Yoichi Nakanishi, Mayu Inagaki, and Shunsuke Wakami in our laboratory. Two previously described independent transgenic lines expressing yeast-soluble PPase IPP1 (*AVP1_pro_::IPP1* #8-3 and *AVP1_pro_::IPP1* #17-3 in the *fugu5-1* mutant background) were used (Ferjani et al., 2011). In addition, the uncoupling variants of *Arabidopsis* H^+^-PPase (U_1_49 and U_2_128) were introduced to *fugu5-3* under the control of the *AVP1* promoter (Asaoka et al., 2016), and the obtained transgenic lines were used.

A modified MGRL culture medium (MGRL^Am^) was prepared to examine the effects of NO_3_^-^ and NH_4_^+^ ions on plant growth. The basal MGRL culture medium for the gel plates contained 1.5 mM NaH_2_PO_4_, 0.26 mM Na_2_HPO_4_, 1.5 mM MgSO_4_, 2.0 mM Ca (NO_3_)_2_, 3.0 mM KNO_3_, 12 μM Fe (III)-EDTA, 10 μM MnSO_4_, 30 μM H_3_BO_3_, 1.0 μM ZnSO_4_, 1.0 μM CuSO_4_, 24 nM (NH_4_)_6_Mo_7_O_24_, 130 nM CoCl_2_, 2% sucrose, and 0.4% gellan gum (Naito et al., 1994). The MGRL^Am^ culture medium contained 3.0 mM NH_4_Cl and 3.0 mM KCl instead of 3.0 mM KNO_3_ and consisted of 1.5 mM NaH_2_PO_4_, 0.26 mM Na_2_HPO_4_, 1.5 mM MgSO_4_, 2.0 mM Ca (NO_3_)_2_, 12 μM Fe (III)-EDTA, 10 μM MnSO_4_, 30 μM H_3_BO_3_, 1.0 μM ZnSO_4_, 1.0 μM CuSO_4_, 24 nM (NH_4_)_6_Mo_7_O_24_, 130 nM CoCl_2_, 2% sucrose, and 0.4% gellan gum. The MGRL^Am^ culture medium contained 4 mM NO_3_^-^ and 3 mM NH_4_^+^.

### Morphological Observations

Whole plants were observed and photographed using a stereoscopic microscope (SZ61; Olympus, Tokyo, Japan) equipped with a CCD camera (DP50; Olympus).

### Scanning Electron Microscopic Observations

For the scanning electron microscopic (SEM) observations, whole plantlets or single leaves were collected and fixed overnight in formalin–acetic acid–alcohol (4% formalin, 5% acetic acid, 50% ethanol) at room temperature. The fixed specimens were dehydrated in an ethanol series [50, 60, 70, 80, 90, 95, 99.5, and 100% (v/v); 60 min per step] and stored overnight in 100% (v/v) ethanol at room temperature. Ethanol was replaced with 3-methylbutyl acetate and the samples were dried in a critical-point dryer (JCPD-5; JEOL, Tokyo, Japan), sputter-coated with gold-palladium using an anion sputter (JFC-1100; JEOL), and examined under an S-3400N scanning electron microscope (Hitachi, Tokyo, Japan) as described previously (Maeda et al., 2014).

### Confocal Laser Scanning Microscopic Observations

Confocal laser scanning microscopic (CLSM) observations were conducted using an upright FV1000-D confocal laser scanning microscope (Olympus). Excitation wavelength and transmission range for emission were 473 nm/485–560 nm for mGFP (Segami et al., 2014). The images were obtained using Olympus FluoView software and an UPLSAPO10X objective lens (Olympus).

### Trypan Blue Staining

Shoots were stained with trypan blue for 3 min at 90°C to visualize dead cells. The trypan blue solution contained 10 ml lactic acid, 10 ml glycerol, 10 ml H_2_O, 10 g phenol, 40 ml ethanol, and 10 mg trypan blue (Shirasu et al., 1999; Kobae et al., 2006). The stained specimens were rinsed with chloral hydrate solution (2.5 g/ml H_2_O) overnight at room temperature prior to microscopic observation.

### Quantification of Phosphate and Pyrophosphate in Plants

Shoots from 20-day-old plants were rinsed three times with pure water, weighed, and then homogenized in two volumes of 20% trichloroacetate. After incubation for 10 min at 4°C, the homogenates were placed in 1.5-ml microcentrifuge tubes before centrifugation for 10 min at 15,000 rpm. The supernatant fractions were collected, diluted 10 times with pure water, and then subjected to phosphate quantification using a Phosphor-C reagent kit (Wako Pure Chemicals).

Shoots from 2-week-old plants were rinsed three times with pure water, weighed, and then frozen in liquid nitrogen. The shoots were homogenized to powder in liquid nitrogen with a mortar and pestle. The tissue powder was suspended with three volumes of pure water, heated at 85°C for 15 min, and then centrifuged for 10 min at 15,000 rpm. The supernatant fractions were collected and re-centrifuged for 10 min at 40,000 rpm. The obtained supernatants were diluted with pure water and subjected to PPi assay using a PPi Assay Kit II (Abcam, Cambridge, UK) according to the manufacturer’s instructions. Fluorescence was monitored with an EnSpire plate reader set at 370 nm for excitation and 470 nm for emission (Perkin Elmer, Waltham, MA, USA).

### Statistical Analysis of the Data

To determine organ fresh weights and for quantification of phosphate and PPi, data from at least three independent experiments were averaged, and the values were subjected to statistical analyses using Student’s *t*-test, Tukey’s honest significant difference test, prop. test or Pearson’s chi-squared test.

## Results

### Atrophic Phenotype of *fugu5* Mutant Leaves Grown on Ammonium-Free Culture Medium

**FIGURE 1:**
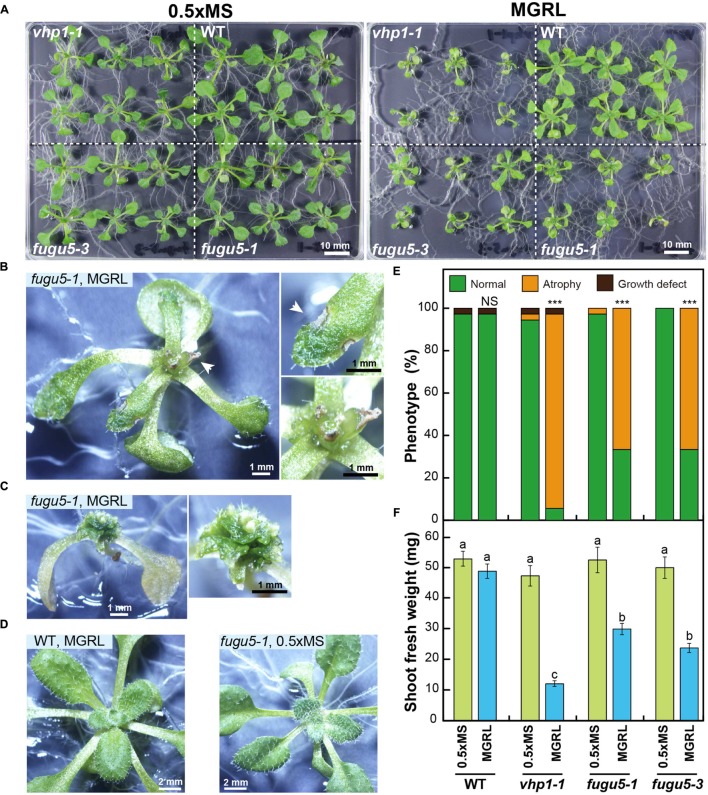
**Leaf atrophy occurs in *fugu5* mutants grown on MGRL culture medium.** **(A)** The H^+^-PPase loss-of-function mutants (*fugu5-1*, *fugu5-3*, and *vhp1-1*) were grown on MS or MGRL plates for 16 days and photographed. **(B-E)** Enlarged images of *fugu5-1* and WT plants taken by a stereoscopic microscope. **(B)** *fugu5-1* grown on an MGRL plate (left). Rosette leaf (right, upper) and a shoot top (right, lower) showing atrophy (arrowheads). **(C)** Severe atrophy of the leaf and shoot of *fugu5-1* grown on an MGRL plate. **(D)** WT grown on an MGRL plate. **(E)** *fugu5-1* grown on an MS plate. (**F**, upper graph) WT and mutant plants were phenotypically sub-classified into three groups: normal plants (green), plants with leaf atrophy (dark yellow), and plants with growth defects (dark brown). The frequencies of these phenotypes are shown (*n* = 36). Asterisks indicate significant differences in the proportion of atrophied plants between MS and MGRL plates at ^∗∗∗^*P* < 0.001 [prop.test; R ver. 3.1.2 (R Core Team, 2014)]. NS, not significant. (**F**, lower graph) Shoot fresh weight. Letters indicate groups divided by Tukey’s honest significant difference test [R ver. 3.1.2 (R Core Team, 2014)]. Error bars show SEs.

In this study, several kinds of culture medium were used for comparative analyses of the potential effects of growth medium composition on plant growth. Surface-sterilized seeds of the WT, *fugu5-1*, *fugu5-3*, and *vhp1-1* mutants were sown onto gellan gum plates and supplemented with MS salt mixture or MGRL nutrients, and their postgerminative growth phenotype was monitored carefully. Surprisingly, severe phenotypic defects were present in the *fugu5-1*, *fugu5-3*, and *vhp1-1* mutants grown in MGRL, but not in those grown on the MS plates (**Figures 1A–E**). On the MGRL plates, the development of most rosette leaves in the *fugu5-1* and *fugu5-3* mutants was arrested, and they were extremely small compared with the WT (**Figure 1A**, right). In this context, the extremely small rosette leaf phenotype will be referred to as leaf atrophy (**Figures 1B,C**). Whereas most of these mutant seedlings had relatively well-developed leaves (**Figure 1B**), a small number of them exhibited more severe phenotypes, with rosette leaves that failed to expand (**Figure 1C**). Even in seedlings showing severe leaf atrophy, the cotyledons were well developed and had a normal shape and size. To discriminate leaf atrophy from growth defects for the purposes of this study, we considered non-germinated seeds and very small seedlings without leaf atrophic symptoms to have growth defects. Based on this classification, the frequency of leaf atrophy was significantly higher in the *vhp1-1*, *fugu5-1*, and *fugu5-3* mutants grown on MGRL plates, whereas almost all plants grew normally on the MS plates (**Figure 1F**, upper graph). Furthermore, shoot fresh weights of the three mutant alleles were less than 60% that of the WT (**Figure 1F**, lower graph). As leaf atrophy was observed specifically and consistently in all three independent mutant alleles, we concluded that this phenotype was due to the loss of function of H^+^-PPase.

### Cell Death in the Basal Regions of Mutant Leaf Blades

**FIGURE 2:**
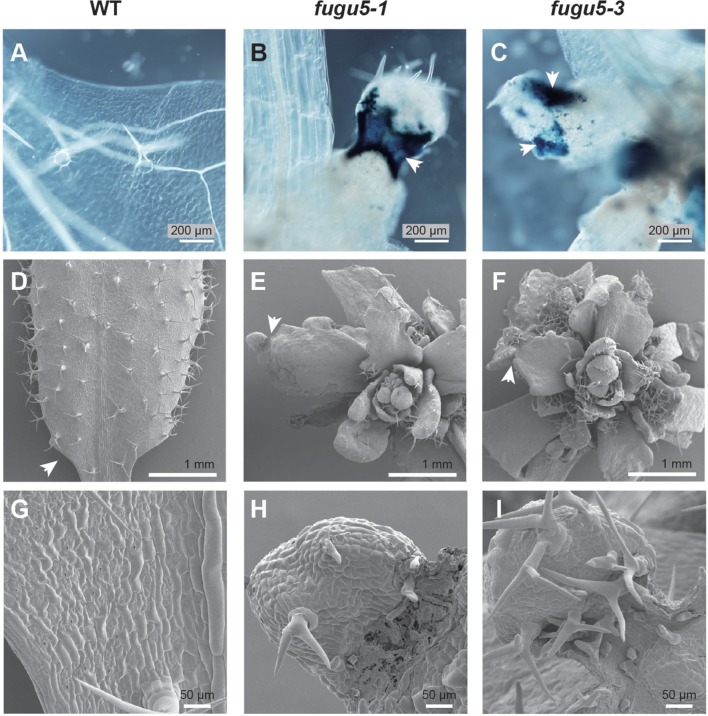
**Cell viability test with trypan blue shows cell death in the basal regions of leaves of *fugu5* grown on MGRL culture medium.** WT, *fugu5-1*, and *fugu5-3* plants were grown on MGRL plates for 2 weeks **(A-C)** or 3 weeks **(D-I)**. The rosette leaves of WT **(A)**, *fugu5-1* **(B)**, and *fugu5-3* **(C)** were stained with trypan blue to test cell viability, as described in the Materials and Methods section. Arrowheads in **(B,C)** indicate the regions stained with trypan blue, where necrosis occurred. The rosette leaves of WT **(D,G)**, *fugu5-1* **(E,H)**, and *fugu5-3* **(F,I)** plants were observed by scanning electron microscopy. Arrows in **(D-F)** indicate the basal regions (leaf blade–petiole junctions) of the rosette leaves, with enlarged images shown in the lower panels **(G-I)**.

Next, to ascertain whether the trophic symptoms in the leaves reflected a cell death phenotype, seedlings of *fugu5* mutants grown on MGRL culture medium were stained with trypan blue, which is retained in dead cells. Importantly, the *fugu5-1* and *fugu5-3* mutants grown on MGRL plates showed strong dark-blue staining in the basal regions of the rosette leaf blades, which may include parts of the petioles (**Figures 2B,C**). As expected, no such dark-blue staining was detected in the WT (**Figure 2A**).

To gain further insight into tissue organization in the atrophic zones, rosette leaves of WT (**Figures 2D,G**), *fugu5-1* (**Figures 2E,H**), and *fugu5-3* (**Figures 2F,I**) were examined by SEM. WT rosette leaves displayed normal cell shapes and tissue organization (**Figure 2D**), whereas *fugu5-1* rosette leaf cells and tissues in the basal region (leaf blade–petiole junction) were smaller, disorganized, and shrunken (**Figures 2E,H**). Consistently, the *fugu5-3* leaves displayed similar morphological defects to *fugu5-1* (**Figures 2F,I**). Taken together, these results strongly suggest that leaf atrophy in the *fugu5* mutants was due to cell death at the rosette leaf blade–petiole junction, also known for its particularly high proliferative activity (Ichihashi et al., 2011; Tsukaya, 2014).

The observed damage in the young rosette leaves led us to examine the expression of H^+^-PPase (*VHP1* gene) in the tissues. When a *VHP1pro::VHP1-mGFP* construct (Segami et al., 2014) was introduced into WT, green fluorescence of H^+^-PPase-mGFP was detected clearly in the first leaves of 7-day-old seedlings (**Supplemental Figure S1**).

### Ammonium in the Culture Medium Prevents Leaf Developmental Damage

**FIGURE 3:**
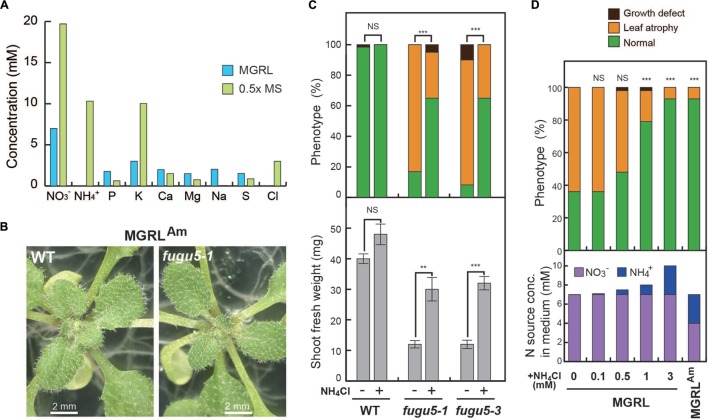
**Leaf atrophy and growth defects of *fugu5* grown on MGRL culture medium are recovered by the addition of NH_4_^+^.** **(A)** Macronutrient contents in the MGRL culture medium (light blue) and 0.5 × MS salt medium (light green). **(B)** Stereoscopic microscope images of WT and *fugu5-1* grown on the MGRL^Am^ culture medium, a modified MGRL supplemented with 3 mM NH_4_Cl and 3 mM KCl. **(C)** WT and mutants were grown on MGRL (NH_4_Cl,-) or MGRL^Am^ (NH_4_Cl, +) plates for 3 weeks. Upper graph; phenotype distribution: normal plants (green), plants with leaf atrophy (dark yellow), and plants with growth defects (dark brown; *n* = 54–60). Lower graph; fresh weights of WT and mutant shoots grown on MGRL or MGRL^Am^ plates. **(D)** Effect of NH_4_^+^ on recovery of growth defects of *fugu5-1* mutants. *fugu5-1* mutants were grown on MGRL culture medium supplemented with NH_4_Cl at the indicated concentrations. In this experiment, only NH_4_Cl was added and all other elements were unchanged. The frequencies of three phenotypic categories are shown in the upper graph (*n* = 42). Concentrations of NO_3_^-^ (purple) and NH_4_^+^ (dark blue) are shown for each culture medium. Error bars show the SEs. Asterisks indicate significant differences at ^∗∗^*P* < 0.01 and ^∗∗∗^*P* < 0.005 compared with plants grown on MGRL plates [prop.test on upper graphs of **(C,D)**; Student’s *t*-test on lower graph of **(C)**; R ver. 3.1.2 (R Core Team, 2014)]. NS, not significant.

The marked differences observed in the growth of mutants in two different culture media (MS and MGRL) led us to compare their composition. Whereas the MGRL culture medium contains nitrate [7 mM; 2 mM Ca (NO_3_)_2_ and 3 mM KNO_3_] as the sole nitrogen source and a negligible amount of ammonium (144 nM), the 0.5 × MS medium contains both nitrate (20 mM) and ammonium (10 mM; **Figure 3A**). To test the effect of ammonium, an MGRL^Am^ culture medium containing 3 mM NH_4_Cl, 3 mM KCl, and no KNO_3_ was prepared. The MGRL^Am^ medium contained 3 mM NH_4_^+^ and 4 mM NO_3_^-^. *fugu5-1* and *fugu5-3* mutants grew well in this medium, and the number of plants with leaf atrophy was markedly reduced (**Figures 3B,C**, upper graph in 3C). The fresh weights of mutant plants were approximately 75% that of the WT, even on the MGRL^Am^ plates (**Figure 3C**, lower graph). Next, we aimed to determine the threshold concentration of NH_4_^+^ needed to prevent leaf atrophy (**Figure 3D**). Our results showed marked recovery at NH_4_Cl concentrations exceeding 1 mM, with a concentration of at least 0.5 mM required (**Figure 3D**).

The MGRL^Am^ medium contained 3 mM NH_4_Cl and 3 mM KCl instead of 3 mM KNO_3_. Thus, we examined whether an additional 6 mM Cl^-^ could prevent leaf atrophy. The results indicated clearly that the addition of KCl or NaCl (6 mM each) did not contribute to the recovery of leaf atrophy (**Supplemental Figure S2A**). Next, sulfate salt [1.5 mM (NH_4_)_2_SO_4_] was used instead of chloride salt (NH_4_Cl) and 1.5 mM K_2_SO_4_ was added to maintain the K^+^ concentration. Importantly, leaf atrophy was reduced significantly in *fugu5-1* and *fugu5-3* mutants grown on the MGRL^Am^ culture medium (**Supplemental Figure S2B**). Taken together, these results strongly suggest that the presence of ammonium ions in the culture medium is required to protect *fugu5* mutant rosette leaves from atrophic damage.

WT, *fugu5-1*, and *fugu5-3* plants did not grow in the nitrate-free culture medium that contained 7 mM NH_4_Cl as the sole nitrogen source (**Supplemental Figure S3**). This result indicates that leaf atrophy caused by ammonium on the *fugu5* mutants depends on the presence of nitrate.

### Physical Contact of Rosette Leaves with the Culture Medium Increases Phosphate Levels and Leaf Atrophy in *fugu5*

**FIGURE 4:**
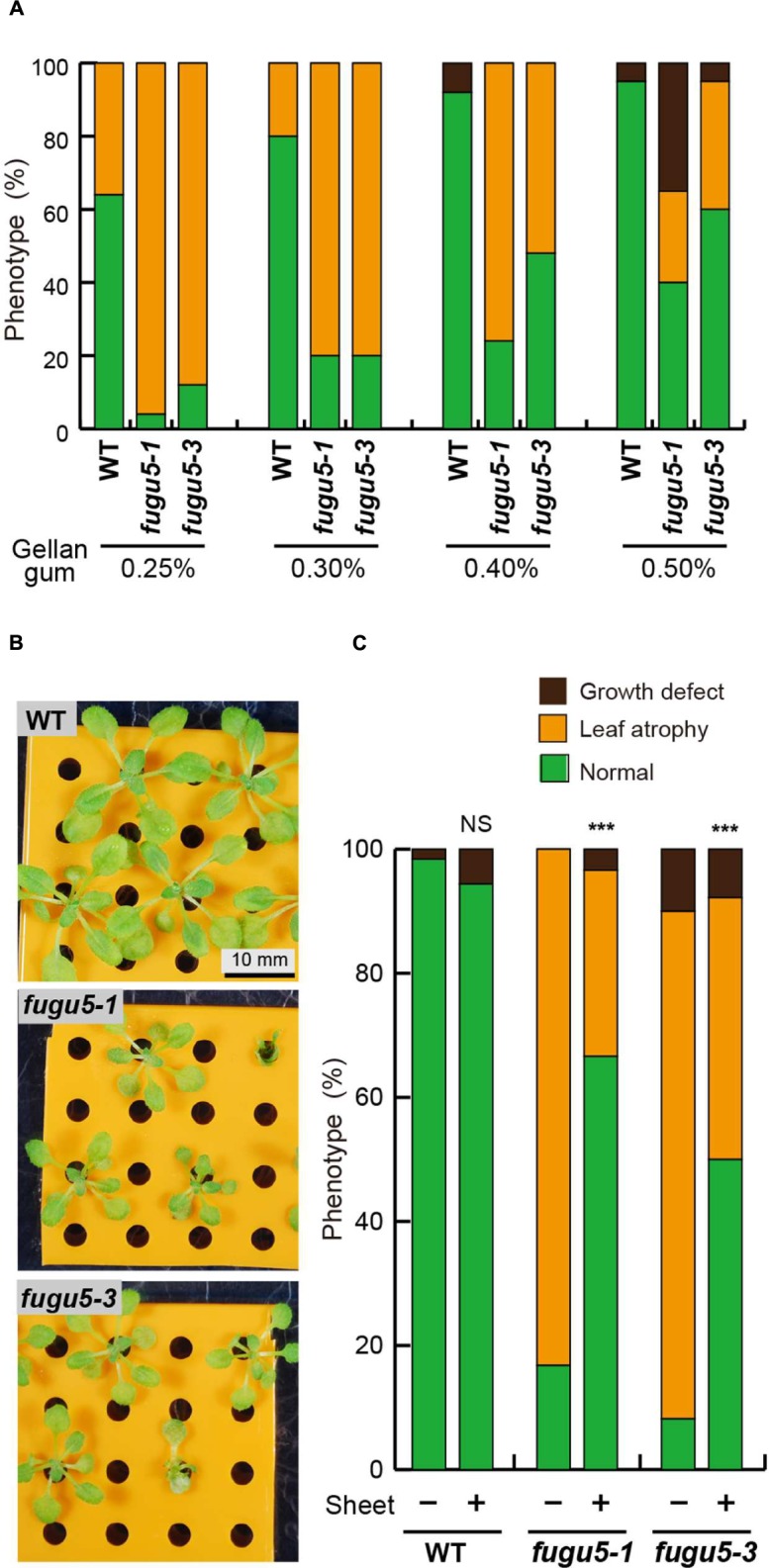
**Physical contact with the MGRL culture medium enhances leaf atrophy.** **(A)** WT and *fugu5* mutants were grown on MGRL plates with the indicated concentrations of gellan gum. The phenotypic distribution for each line is shown (*n* = 25). **(B)** WT and *fugu5* mutants were grown on MGRL plates with 0.4% gellan gum, which were set with a porous plastic sheet to prevent direct contact of leaves with the culture medium. **(C)** The frequencies of three categories of phenotype for each line grown on MGRL culture medium with (+) or without (-) the sheet. Asterisks indicate significant differences at ^∗∗∗^*P* < 0.001 compared with plants grown on MGRL plates without a plastic sheet [prop.test: R ver. 3.1.2 (R Core Team, 2014)] (*n* = 60). NS, not significant.

The cotyledons and rosette leaves of WT and *fugu5* mutants are curled, and their tips were often in direct contact with the surface of the culture medium. First, the effect of MGRL plate hardness on the phenotype of *fugu5* mutants was investigated because seedlings stick easily to the surface of the culture medium when the gel is relatively soft. Interestingly, the frequency of leaf atrophy at 0.25% gellan gum concentration, which produced soft gels, was significantly higher than observed on harder gels (0.3–0.5%; **Figure 4A**). Based on these findings, we attempted to prevent leaf–culture medium contact completely by laying down porous, thick plastic sheets (**Figure 4B**). The frequency of leaf atrophy in the mutants was decreased significantly when the leaves were not in direct contact with the culture medium (**Figure 4C**), although overall growth of the *fugu5* mutants was delayed under the same growth conditions (**Figure 4B**). These results support our hypothesis and suggest that physical contact of *fugu5* leaves with the MGRL culture medium somehow triggered leaf atrophy.

**FIGURE 5:**
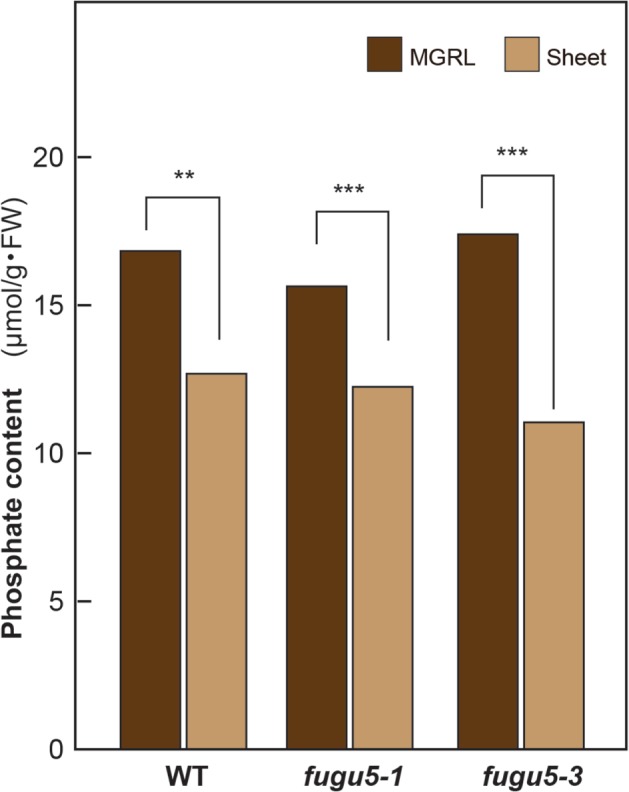
**Contact of rosette leaves with the culture medium increases the phosphate contents of plant shoots.** WT, *fugu5-1*, and *fugu5-3* were cultivated on MGRL plates for 3 weeks with or without plastic sheets that prevented contact with culture medium. The phosphate contents of shoot extracts were determined as described in the Materials and Methods section. Asterisks indicate significant differences at ^∗∗^*P* < 0.01 and ^∗∗∗^*P* < 0.005 compared with plants grown without plastic sheets (Student’s *t*-test; *n* ≥3).

In this case, some elements in the culture medium were likely absorbed through the leaf tips. To further examine this possibility, the phosphate contents of 3-week-old plantlets of WT and both *fugu5* mutant alleles were quantified. Interestingly, the phosphate contents in the WT, *fugu5-1*, and *fugu5-3* plants were reduced significantly by up to 76, 77, and 64%, respectively, when grown using the plastic sheets that prevented contact with the culture medium (**Figure 5**).

### Specific Removal of PPi from the *fugu5* Mutant Background Restored Normal Growth

**FIGURE 6:**
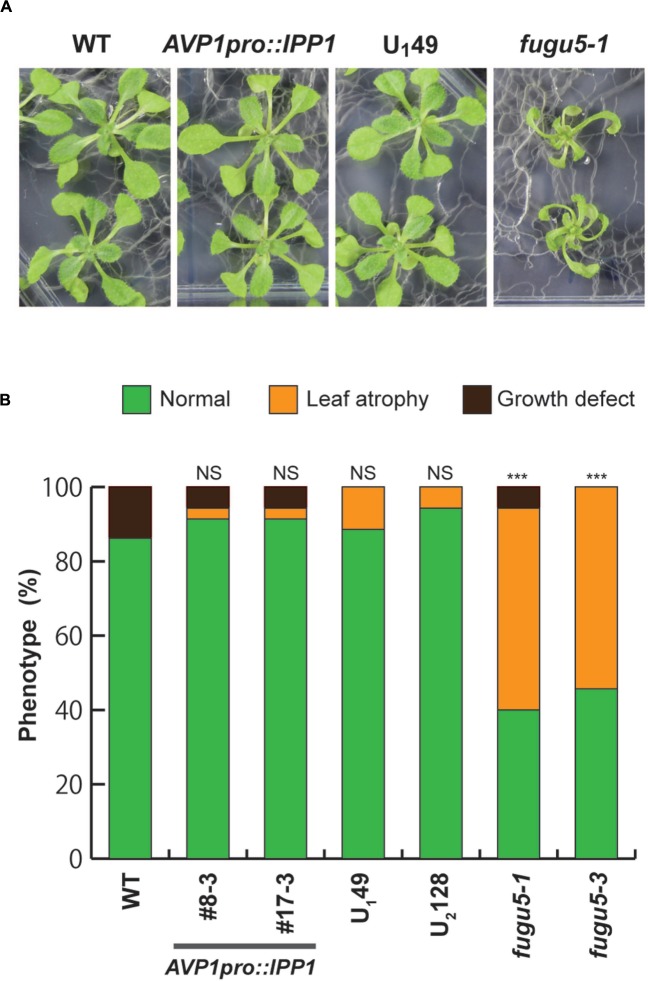
**Leaf atrophy and necrotic symptoms are rescued by the introduction of yeast-soluble PPase and the uncoupling-type H^+^-PPase into *fugu5* mutants.** **(A)** Yeast-soluble PPase (IPP1) (Ferjani et al., 2011) and the uncoupling variants of *Arabidopsis* H^+^-PPase (U_1_49 and U_2_128) (Asaoka et al., 2016) were introduced to *fugu5-1* and *fugu5-3*, respectively, under the control of the *AVP1* promoter. Plants were grown on MGRL plates for 3 weeks before being photographed. **(B)** WT, *fugu5-1*, *fugu5-3* and transgenic plants were grown on MGRL plates for 3 weeks. Phenotypic distribution: normal plants (green), plants with leaf atrophy (dark yellow), and plants with growth defects (dark brown). The frequencies of these phenotypes are shown (*n* = 25). Asterisks indicate significant differences at ^∗∗∗^*P* < 0.001 compared with plants grown on MGRL plates (Pearson’s chi-squared test). NS, not significant.

The plant vacuolar H^+^-PPase functions as a proton pump coupled with hydrolysis of PPi (Maeshima, 2000). All H^+^-PPase loss-of-function mutants in this study lacked PPi hydrolysis and proton pumping activities (Ferjani et al., 2011), and it was unclear which of these functions was responsible for the leaf-atrophic phenotype. We examined the effect of complementation of PPi hydrolysis activity by introducing a yeast soluble–type PPase (IPP1) into the *fugu5-1* background. IPP1 was introduced under the control of the *VHP1*/*AVP1* promoter (*AVP1_pro_::IPP1*; Ferjani et al., 2011). Therefore, these transgenic lines were able to hydrolyze PPi, but lacked the proton pumping activity of the vacuolar H^+^-PPase. Importantly, our results showed that the growth of two independent *AVP1_pro_::IPP1*transgenic lines (*#8-3* and *#17-3*) on the MGRL culture medium was indistinguishable from that of the WT (**Figure 6A**), and no leaf atrophy was observed (**Figure 6B**).

In parallel to the *AVP1_pro_::IPP1* transgenic lines, uncoupling variants of *Arabidopsis* H^+^-PPase were generated that were able to hydrolyze PPi efficiently, but which failed to translocate protons across the tonoplast (Asaoka et al., 2016). These uncoupling variants of H^+^-PPase were able to rescue the morphological defects of the *fugu5* mutant (Asaoka et al., 2016). To confirm our findings, U_1_49 and U_2_128 (two independent transgenic lines expressing uncoupling H^+^-PPase variants in the *fugu5-3* background) were grown on MGRL plates, and their phenotypes were analyzed as described above. Consistently, the U_1_49 plant shoots were indistinguishable from the WT (**Figure 6A**), and no leaf atrophy was observed in the U_1_49 and U_2_128 lines (**Figure 6B**). These results demonstrated clearly that the observed leaf-atrophic phenotype of *fugu5* on the ammonium-free culture media was caused by the loss of the PPi-hydrolyzing activity of the H^+^-PPase.

To further confirm our findings, the PPi contents of the WT, *AVP1_pro_::IPP1*, *fugu5-1*, and *fugu5-3* were quantified. The PPi contents in plants grown on the MGRL^Am^ culture medium were equivalent in all four genotypes (**Supplemental Figure S4B**). However, PPi levels were significantly higher in the *fugu5* mutants than in the WT (**Supplemental Figure S4B**). As expected, the *AVP1_pro_::IPP1* lines showed normal levels of PPi, similar to that of the WT. Taken together, our findings indicate that PPi levels were significantly higher in *fugu5* mutants than in the WT when grown on ammonium-free culture medium.

## Discussion

Recent studies have revealed that the PPi-hydrolyzing activity of vacuolar H^+^-PPase is essential for the normal development of cotyledons and hypocotyls when plants are germinated on sucrose-free culture medium (Ferjani et al., 2011, 2014a,b). In our study, H^+^-PPase loss-of-function mutants (*fugu5*s and *vhp1-1*) displayed drastic morphological and novel developmental defects when grown on ammonium-free culture medium. These results suggest that the mutants are not able to grow normally when nitrate is the sole nitrogen source in the medium. The overall growth of the mutant shoots was markedly compromised (**Figures 1F** and **3C**), which resulted in the developmental arrest of rosette leaves (**Figures 1A,C**). Trypan blue staining patterns revealed that the leaf-atrophic phenotypes were the result of local cell death in the tissues at the leaf blade–petiole junctions of young developing rosette leaves (**Figure 2**). The arrested growth of the mutant rosette leaves also could be interpreted as a result of a premature arrest at an early vegetative growth stage that was triggered specifically by the lack of ammonium in the culture media. The particular susceptibility of the blade–petiole junction to atrophic damage might reflect the abundance of H^+^-PPase in this region, known for its high cell proliferation activity (Ichihashi et al., 2011; Tsukaya, 2014). Indeed, our CLSM observations revealed extensive expression of H^+^-PPase during the cell proliferation stage in 7-day-old seedlings expressing *VHP1_pro_:VHP1-mGFP* (**Supplemental Figure S1**; Segami et al., 2014). A high level of H^+^-PPase in these tissues hints at a key role in scavenging cytosolic PPi generated by active macromolecule biosynthesis for active cell proliferation and cell growth.

The developmental defects described in this study occurred in *fugu5*s and *vhp1* seedlings grown on MGRL plates. The MGRL culture medium contains sufficient amounts of essential elements (Naito et al., 1994), and is used widely in laboratories. On the other hand, the MS culture medium was developed originally for plant tissue culture and is now used for seed germination and suspension-cell culture. The above-mentioned phenotype of *fugu5*s and *vhp1-1* was not observed on the MS plates. The major difference between the MGRL and MS culture media lies in the concentrations of nitrate and ammonium. The MGRL culture medium contains only 7 mM NO_3_^-^ and a trace amount of NH_4_^+^, whereas the 0.5 × MS contains 20 mM NO_3_^-^ and 10 mM NH_4_^+^ (**Figure 3A**). Ammonium and nitrate are the major nitrogen sources for plants. Although nitrate levels are generally higher than ammonium levels in soil, most plants, including *Arabidopsis*, have the ability to use both nitrate and ammonium as nitrogen sources (Stitt et al., 2002; Gruber and Galloway, 2008; Robertson and Vitousek, 2009).

This study revealed that the addition of >1 mM ammonium to the standard MGRL culture medium significantly prevented the leaf-atrophic phenotype of *fugu5*s (**Figure 3D**). This prevention was achieved by NH_4_Cl or (NH_4_)_2_SO_4_, but not by other ions, such as K^+^, Cl^-^, and SO_4_^2-^ (**Supplemental Figure S2**). The MGRL^Am^ culture medium containing 3 mM NH_4_Cl and 2 mM Ca (NO_3_)_2_ was also sufficient for normal growth of *fugu5*s. The concentration of NH_4_^+^ required for normal growth of the mutants was relatively high, suggesting that ammonium is used in plants as a nutrient, rather than as a signaling molecule. When the roots absorb NO_3_^-^, cells in these tissues convert it to NH_4_^+^ and then to amino acids through several metabolic steps (Stitt et al., 2002). The metabolic reduction of NO_3_^-^ to NH_4_^+^ requires energy consumption *in vivo*. Thus, ammonium has an advantage from a metabolic energy point of view. Nevertheless, the WT and *fugu5* mutants showed severe growth defects when ammonium was used as the sole nitrogen source (**Supplemental Figure S3A**). A possible explanation is that excess amount of ammonium may be toxic to living cells because of its penetration of membranes and collapse of the membrane potential. Therefore, the *fugu5* mutants require not only ammonium, but also nitrate for their optimal growth.

In relation to the above-described phenotypes, we examined whether the absence of ammonium could change PPi metabolism in the *fugu5* mutant cells. When the lack of PPi hydrolysis function in *fugu5* was complemented by yeast-soluble PPase and the uncoupling-type H^+^-PPase, the leaf-atrophic phenotype was simultaneously and markedly suppressed in plants grown on the MGRL plates (**Figure 6**). Furthermore, *fugu5* mutants accumulated more PPi in the shoots than did the WT when grown on the MGRL culture medium (**Supplemental Figure S4**). Thus, the presence or absence of ammonium in the medium likely affects the concentration of PPi in the mutant cells. In addition, contact of cotyledons and/or rosette leaves with the medium enhanced leaf atrophy in plants grown on MGRL plates. Therefore, some minerals, which are probably taken up through the hydathodes at the leaf tips, may somehow contribute to enhanced leaf atrophy. Hydathodes exist in the tips and margins of leaves, and usually secrete water. Under specific conditions, components such as glutamine are also extruded through the hydathodes (Pilot et al., 2004). Hydathodes contain several ion transporters, including phosphate, sulfate, and potassium transporters (Lagarde et al., 1996; Mudge et al., 2002; Shibagaki et al., 2002; Wang et al., 2004; Nagai et al., 2013), which are thought to enable re-absorption of mineral ions released through guttation.

Previous studies revealed that *fugu5* accumulated unusually high levels of PPi, and that the expression of IPP1 in the *fugu5* background (in the *AVP1_pro_::IPP1* lines) was sufficient to rescue its developmental defects, irrespectively, of the growth conditions (Ferjani et al., 2011, 2014a). Under such circumstances, phosphate might be imported from the medium (which contains phosphate at 1.76 mM) into the leaves through phosphate transporters in the hydathode plasma membrane. Such uptake of phosphate might suppress PPi hydrolysis by H^+^-PPase and soluble PPase(s), as the latter reaction releases inorganic phosphate. In addition, whereas PPi levels in the WT are strictly controlled, such H^+^-PPase–mediated regulation is completely lost in the *fugu5* mutants. Thus, the increased PPi in the mutants (**Supplemental Figure S3**) should inhibit the biosynthetic reactions of macromolecules, such as DNA, RNA, proteins, and cellulose (Maeshima, 2000; Heinonen, 2001; Bertoni, 2011; Ferjani et al., 2014a,b). As a result, cell proliferation and subsequent cell elongation might be strongly compromised. Alternatively, the inhibition of cell wall synthesis due to the shortage of cell wall material may also affect the physical strength of the cells, which, in extreme cases, may alter their role as a physical barrier and affect the selective uptake of some component in the culture media.

The present study showed that leaf atrophy phenotype of *fugu5* mutants in the ammonium-free culture medium was caused by dysfunction of PPi hydrolysis. Although these scenarios enable speculation about the mechanism(s) of PPi-triggered leaf atrophy, it should be noted that the presented linkage between the PPi-hydrolysis by H^+^-PPase and plant response to nitrogen source has at present no clear mechanistic causal basis. Further experiments are needed that address, for example, the transcriptional and functional changes in PPi-utilizing enzymes, such as PFP, UGPase, PPDK, and soluble PPases, under both nutritional regimes. In conclusion, this study revealed interesting phenotypes specific to the H^+^-PPase loss-of-function mutants which, to our knowledge, are novel. Future studies should be carried out, with priority given to the examination of the relationship between PPi hydrolysis and leaf atrophy.

## Author Contributions

MF co-coordinated the project, contributed to phenotyping, analyzed the data, and drafted the manuscript; SS conducted the association analysis; SG conducted the SEM observations; AF provided the *fugu5* mutants and the *AVP1_pro_::IPP1* transgenic lines, and contributed to the manuscript; TT conducted the PPi quantification; YN isolated the *vhp1* mutant line and gave advice on the experiments; MA prepared the mutant lines expressing the uncoupling type H^+^-PPase; MM conceived and initiated the project, obtained funding, and contributed to the manuscript. All authors read and approved the final manuscript.

## Conflict of Interest Statement

The authors declare that the research was conducted in the absence of any commercial or financial relationships that could be construed as a potential conflict of interest.

## Abbreviations

H^+^-PPase: H^+^-translocating pyrophosphatase
IPP: inorganic pyrophosphatase
PPi: pyrophosphate
PFP: PPi-dependent phosphofructokinase
UGPase: UDP-glucose pyrophosphorylase
PPDK: pyruvate orthophosphate dikinase
