# Supplementary material for: Lack of H+-pyrophosphatase Prompts Developmental Damage in Arabidopsis Leaves on Ammonia-Free Culture Medium
Source: Front Plant Sci. 2016 Jun 10;7:819. doi: 10.3389/fpls.2016.00819 (PMC4901044; doi:10.3389/fpls.2016.00819)
Supplement: Supplementary file 1 [file Data_Sheet_1.PDF]

Supplemental Data

Lack of H^+-pyrophosphatase prompts developmental damage in Arabidopsis leaves on ammonia-free culture medium

Mayu Fukuda [1,a], *Shoji Segami* [1,a,b], *Takaaki Tomoyama* [1], *Mariko Asaoka* [1,2], *Yoichi Nakanishi* [1], *Shizuka Gunji* [2], *Ali Ferjani* [2], *and Masayoshi Maeshima* [1,b]

[1] *Laboratory of Cell Dynamics, Graduate School of Bioagricultural Sciences, Nagoya University, Nagoya 464-8601, Japan,* [2] *Department of Biology, Tokyo Gakugei University, Nukui Kitamachi 4-1-1, Koganei-shi, Tokyo 184-8501, Japan*

Supplemental Figures 1 to 4

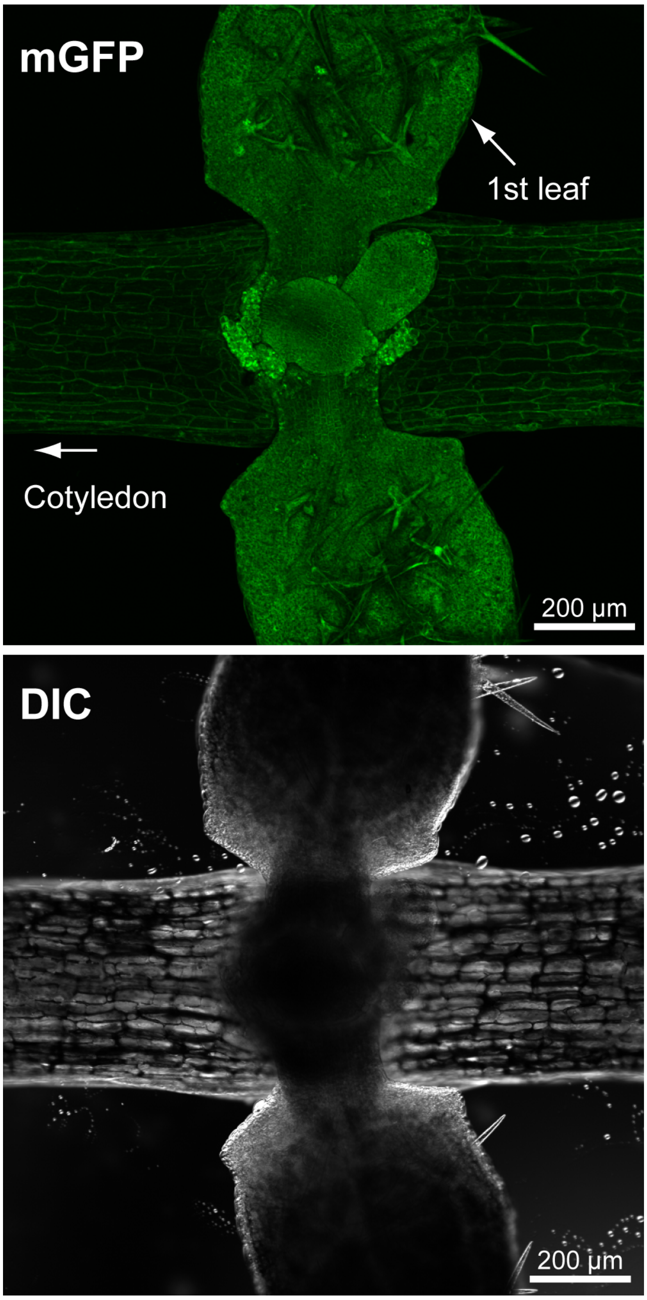

Supplemental Figure 1 | Distribution of H^+-PPase in young leaf. Seven-day-old seedlings expressing *VHP1$_{pro}$::VHP1-mGFP* (line wM-A9) (Segami et al., 2014) that have been grown on MS medium and were observed with an upright FV1000-D confocal laser scanning microscope (Olympus). The images were obtained using Olympus Fluoview software and a UPLSAPO10X objective lens (Olympus). Upper panel (mGFP) shows a green fluorescence image constructed by projection of z-stack images with a 4.23 µm-interval and lower panel an image of differential interference contrast (DIC).

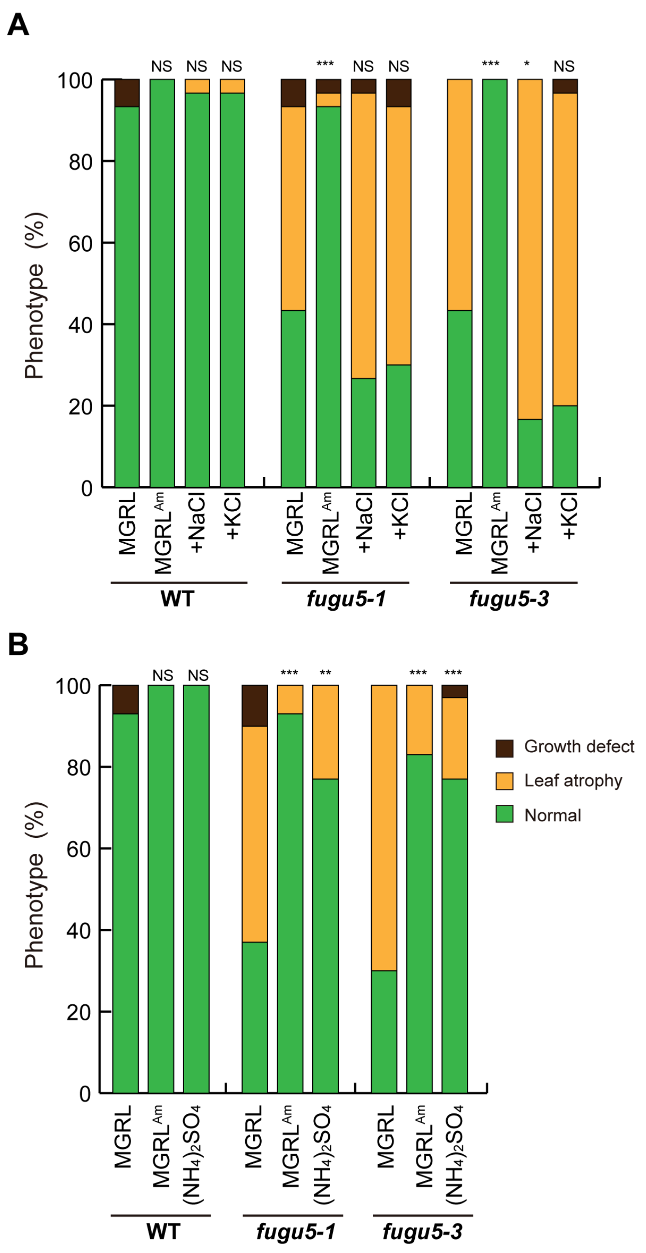

Supplemental Figure 2 | Effect of additional ions in the culture medium on recovery of leaf atrophy of *fugu5* mutants. (A and B) WT and *fugu5* mutants were grown on MGRL and modified MGRL culture media. Experiments for A and B were independently carried out (n = 24 - 40). Asterisks indicate significant differences at $**P < 0.01$ and $***P < 0.005$ compared with plants grown on MGRL plates (Pearson's chi-squared test). The following salts were used as the nitrogen source.

(MGRL): 2 mM $Ca(NO_3)_2$ and 3 mM KNO_3.

($MGRL^{Am}$): 2 mM $Ca(NO_3)_2$, 3 mM NH_4Cl and 3 mM KCl.

(+NaCl): 2 mM $Ca(NO_3)_2$, 3 mM KNO_3 and 6 mM NaCl,

(+KCl): 2 mM $Ca(NO_3)_2$, 3 mM KNO_3 and 6 mM KCl.

($(NH_4)_2SO_4$); 2 mM $Ca(NO_3)_2$, 1.5 mM $(NH_4)_2SO_4$ and 1.5 mM K_2SO_4.

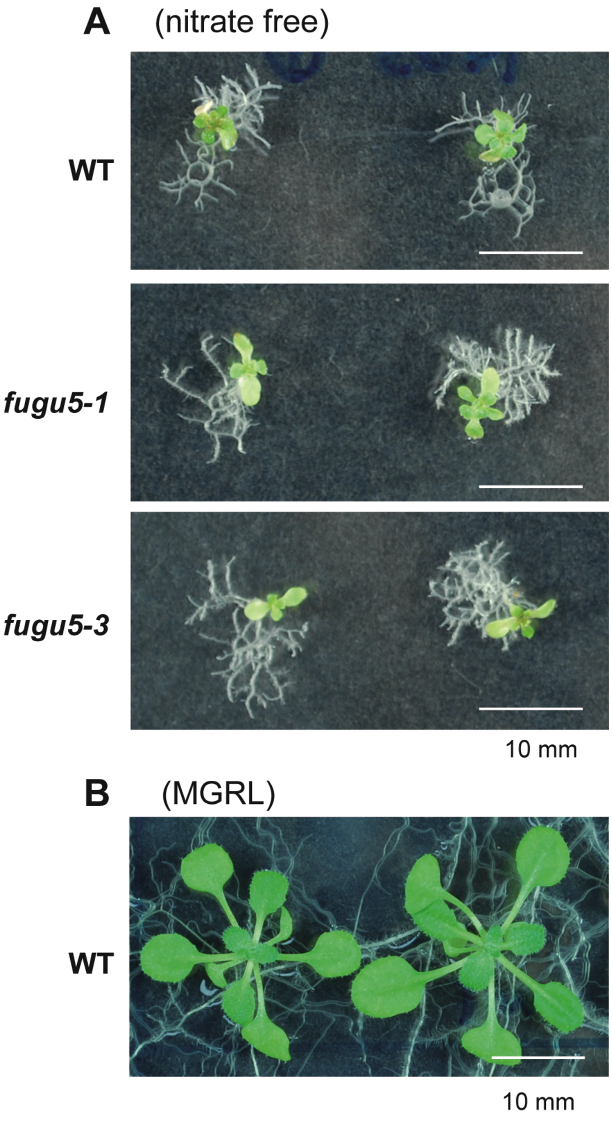

Supplemental Figure 3 | Gross morphology of WT and *fugu5* mutants grown on nitrate-free culture medium. (A) WT, *fugu5-1* and *fugu5-3* were grown for 3 weeks on nitrate-free culture medium, which contained 2 mM $CaCl_2$, 7 mM NH_4Cl and 3 mM KCl instead of 2 mM $Ca(NO_3)_2$ and 3 mM KNO_3 for regular MGRL medium. (B) WT was grown on MGRL plates for 3 weeks as a control.

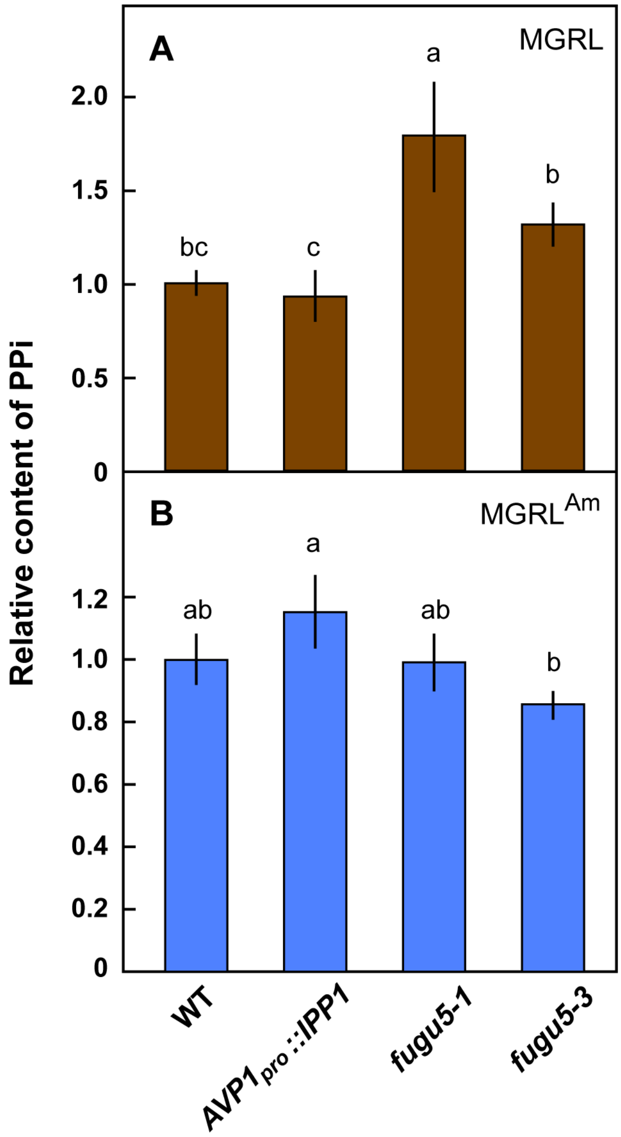

Supplemental Figure 4 | Contents of pyrophosphate in 2-week-old plant shoots. WT, *AVP1*$_{pro}$*::IPP1*, *fugu5-1* and *fugu5-3* were grown for 2 weeks on MGRL (A) and MGRL^{Am} plates (B). Pyrophosphate contents in tissue extracts were determined using a Pyrophosphate Assay kit II as described under the Methods. The content is expressed as relative values to that of WT grown on each culture medium. The PPi contents of WT grown on MGRL and MGRL^{Am} were 1.56 and 1.89 μmol/g fresh weight, respectively. Error bars show SD (n = 3). At least fourteen plants were used per each single measurement. Alphabets indicate significant difference at P < 0.05 compared to WT (Tukey's honest significant difference test).
